# Dermoscopic Analysis of Vascular Malformations and Tumors Based Upon Dominant Vascular Dermoscopic Features: A Retrospective Analysis From a Tertiary Care Center of East India

**DOI:** 10.7759/cureus.26292

**Published:** 2022-06-24

**Authors:** Parvathy Viswan, Biswanath Behera, Madhusmita Sethy, Siddhartha Dash, Aparna Palit, Pavithra Ayyanar

**Affiliations:** 1 Dermatology, Venereology and Leprology, All India Institute of Medical Sciences, Bhubaneswar, Bhubaneswar, IND; 2 Pathology and Laboratory Medicine, All India Institute of Medical Sciences, Bhubaneswar, Bhubaneswar, IND; 3 Dermatology, Venereology and Leprology, All India Institute of Medical Sciences, Kalyani, Kalyani, IND

**Keywords:** vascular malformation, vascular tumor, skin of color, hemoglobin, dermatoscopy, dermoscopy

## Abstract

Background

Cutaneous vascular malformations and tumors comprise a vast group of conditions with variable clinical presentations. It is imperative to differentiate them from nonvascular lesions and from each other as their management and prognosis differ significantly. There is only sparse literature on dermoscopic features of various vascular malformations and tumors, especially from India.

Aim

We aimed to retrospectively study the dermoscopic findings of various vascular malformations and tumors based on their dominant vascular dermoscopic feature.

Method

All the vascular malformations and tumors for which clinical details and clinical and dermoscopic images were available were included in the analysis. The dominant vascular feature(s) was defined as a single or combination of two or more vascular features (in case a single vascular feature does not satisfy the criteria) that constitute more than 75% of the lesions’ vascular features. These included red, purple, blue, black (or any combination) dots, globules, lacunae, structureless area, linear, linear irregular, hairpin, comma, and arborizing vessels.

Results

A total of 52 patients with 68 vascular lesions (22 vascular malformations and 46 vascular tumors) were analyzed. Port-wine stain showed linear irregular vessels with sharp border with or without intervening white structureless area; unilateral nevoid telangiectasia had red dots and globules; angiokeratoma displayed red, reddish-purple to brown lacunae; blue color was seen in venous and glomuvenous malformation and venous lake; a mixed pattern was noted in infantile hemangioma and verrucous hemangioma; a red to reddish-white structureless area was observed in pyogenic granuloma and cherry angioma, and a subungual ill-defined pink structureless area was spotted in subungual glomus tumor.

Conclusion

The dermoscopic features observed in various vascular lesions may overlap; however, the dominant dermoscopic feature along with its color may point to the diagnosis.

## Introduction

Cutaneous vascular malformations and tumors can include a vast group of conditions with variable clinical presentations. Therefore, it is imperative to differentiate them from nonvascular lesions and from each other as their management and prognosis differ significantly. In addition, the development of brown to black color in vascular lesions due to thrombosis can be confused with various pigmented nonvascular tumors such as melanocytic nevus, pigmented basal cell carcinoma (BCC), Bowen’s disease, and seborrheic keratosis, especially in patients with skin of color [1,2]. Dermoscopy is being increasingly used to aid in the diagnosis of various dermatoses by displaying pigmented, non-pigmented, and vascular features. It has been used as a supplementary diagnostic tool that increases the pretest probability of a diagnosis of a dermatosis before pathological examination and at times abates the need for biopsy. There is only sparse literature on dermoscopic features of various vascular malformations and tumors, especially in skin of color [2]. Most studies report various vascular features, but no studies describe dermoscopic differences between various vascular tumors and malformations.

We aimed to retrospectively study the dermoscopic features of various vascular malformations and tumors based on their dominant vascular dermoscopic features.

## Materials and methods

The study was conducted in a tertiary care center in East India after approval from the institute's ethics committee. All the cases of vascular malformations and tumors between August 2018 to August 2021 were assessed for inclusion. Clinical details of the patients were noted from clinical notes and pathological forms. All the clinical and dermoscopic images were retrieved from the departmental archives. Clinical images were photographed using Canon digital camera (PowerShot SX620 HS, Canon, Tokyo, Japan). Dermoscopic images were taken using a DermLite DL4 dermatoscope (DermLite LLC, San Juan Capistrano, CA) in both polarized and nonpolarized mode at 10X magnification attached to a Canon digital camera (PowerShot SX620 HS). The cases of both sex and all age groups with available clinicodemographic details and clinical and dermoscopic images were included in the analysis. The exclusion criteria were the following: lesions with uninterpretable clinical and/or dermoscopic images, patients who received either medical or surgical treatment before the dermoscopic examination, and cases with ambiguous diagnoses.

The dermoscopic vascular features were defined as the features resulting from the presence of (dilated) vessels in the cutaneous lesions, due to the alteration of the blood inside the lumen of the vessels and, or due to the proliferating endothelial cells. Blood spots/hemorrhage were excluded from this group due to the presence of blood outside the vessel's lumen. The vascular features that were analyzed were as follows: red, purple, blue, black (or any combination) dots, globules, lacunae, and structureless area, linear, linear irregular, hairpin, comma, and arborizing vessels. Vascular dots, globules, lacunae, and structureless areas were predefined based on the size as follows: dots (round to oval well-demarcated structures less than 0.1 mm), globule (round to oval well-demarcated structures of size 0.1 mm - 0.2 mm), lacunae (well-demarcated round or oval area of size more than 0.2 mm and less than 2 mm), and structureless areas (an ill-defined area of any size or a well-defined area of size more than 2 mm) [3]. The dermoscopic features were analyzed based on the dominant vascular feature(s), defined as a single or combination of two or more vascular features (in case a single vascular feature does not satisfy the criteria) that constitute more than 75% of the lesions’ vascular features. Other dermoscopic features analyzed were crust, blood spots, comedo-like opening, linear-irregular crypts, keratotic plugging, ulceration, shiny white structures, and multicolored pattern.

All the pathological slides of the included cases were collected from the pathology department. Two independent pathologists reviewed all the available slides for pathological features and immunohistochemistry and agreed upon the original diagnosis. In addition, the corresponding pathological findings of the dermoscopic vascular features were noted.

## Results

A total of 52 patients with 68 vascular lesions were included in the analysis. Of them, 22 were vascular malformations and 46 were vascular tumors. The age of the patients varied from two months to 76 years and of the 52 patients, 22 were female. All of them belonged to the skin phototypes IV, and V.

Of the 22 lesions of vascular malformations, the frequency of various lesions was as follows: Port-wine stain (PWS, n = 5, 22.7%), angiokeratoma (AK) circumscriptum (n = 5, 22.7%), unilateral nevoid telangiectasia (UNT, n = 3, 13.6%), hereditary hemorrhagic telangiectasia (HHT, n = 2, 9.1%), glomuvenous malformation (n = 2, 9.1%) and 1 (4.5%) lesion each of angioma serpiginosum (AS), AK of Mibelli, AK of Fordyce, venous malformation and venous lake. Males and females were equally affected, with five cases having the onset of the disease at birth, five in childhood, three in adolescence, and nine in adulthood.

Of the 46 lesions of vascular tumors, 15 (32.6%) were cherry angioma, followed by pyogenic granuloma (PG, n = 12, 26.1%), infantile hemangioma (IH, n = 9, 19.6%), subungual glomus tumor (n = 3, 6.5%), verrucous hemangioma (VH, n = 2, 4.3%) and 1 (2.2%) lesion each of tufted angioma, partially involuting congenital hemangioma (PICH), non-involuting congenital hemangioma (NICH), glomus tumor and angiosarcoma. Males were slightly more affected with a male to female ratio of 1.3:1. Two cases had the onset of the disease at birth, four in infancy, three in childhood, one in adolescence, and 20 in adulthood.

The dominant vascular feature(s) and other dermoscopic features of vascular malformations and tumors are enlisted in Tables 1-2, respectively.

**Table 1:** Dermoscopic features of vascular malformations Dermoscopic features of vascular malformations

Vascular malformation or tumor	Dominant vascular dermoscopic feature	Other dermoscopic features
Port-wine stain	Linear irregular vessels	Red dots
		Red to reddish-purple globules and structureless area
		White to reddish-white structureless area
Hereditary hemorrhagic telangiectasia	Red to reddish-purple structureless area	White to reddish-white structureless area
		Red dots
Unilateral nevoid telangiectasia	Red dots and globules	Brown structureless area
		Brown network or broken network
Angioma serpiginosum	Linear irregular vessels	Red dots and globules
		Linear vessels
		Brown structureless area
Angiokeratoma circumscriptum	Red, reddish-purple to brown lacunae	Red dots
		Red to reddish-purple globules
		Red to reddish-purple, white to reddish-white, yellow to reddish-yellow structureless area
		White network
		Shiny-white lines
		Comedo-like opening
Angiokeratoma of Mibelli	Red, reddish-purple to brown lacunae	Red dots
		Red to reddish-purple globules
Angiokeratoma of Fordyce	Red to reddish-purple lacunae	Red to reddish-purple globules
		White to reddish-white structureless area
		Shiny white structureless area
Venous malformation	Bluish to bluish-red lacunae and structureless area	Red to reddish-white globules
		Red lacunae
		Brown structureless area
Glomovenous malformation- plaque	Bluish-white to bluish-red structureless area	Red dots and globules
Glomovenous malformation- papular	Red to reddish-purple structureless area	-
Venous lake	Blue, reddish-blue to bluish-purple structureless area	-

**Table 2:** Dermoscopic features of cutaneous vascular tumors Dermoscopic features of cutaneous vascular tumors

Vascular tumors	Dominant vascular dermoscopic feature	Other dermoscopic features
Superficial infantile hemangioma	Red to reddish-purple globules, and lacunae	Red dots
		Shiny white lines
	Red to reddish-white structureless area	Brown dots
Deep infantile hemangioma	White to bluish-white structureless area	Linear irregular vessels
Tufted hemangioma	Skin-colored to light pink structureless area	Cerebriform pattern
Partially involuting congenital hemangioma	Linear irregular vessels	Red dots and red to reddish-purple globules
Noninvoluting congenital hemangioma	Red dots, globules, and lacunae	White network
Pyogenic granuloma	Pink, red to reddish-white structureless area	Red to reddish-purple globules
		Yellow to reddish-yellow structureless area
		Shiny white lines and structureless area
		White scales
		White lines
		Crust
		Blood spots
Cherry angioma	Pink, red to reddish-white structureless area	Red to reddish-purple lacunae
		White lines
		Linear irregular vessels
Verrucous hemnagioma	Red to reddish-purple dots, globules, and structureless area	Red to reddish-purple lacunae
		Pink, red to reddish-white structureless area
		Shiny white line and structureless area
		Rosette
		White scales
		Comedo-like opening with keratotic plugging
		Linear irregular crypts
Glomus tumor	Pink structureless area	-
Subungual glomus tumor	Subungual pink to pinkish-white structureless area	-
Angiosarcoma	Red, reddish-white to reddish-purple structureless area	Ulceration
		Blood spot
		Shiny white structureless area

## Discussion

Vascular malformations are less commonly described lesions, and it is difficult to describe accurate epidemiologic data due to varying nomenclature and definition of congenital vascular malformations in literature. The reported prevalence of congenital vascular malformations is around 1.2%-1.5%. Out of the total, around two-thirds are predominantly venous, one-quarter are complete/partial lymphatic origin and the rest are high flow malformations. There is no gender predilection and they appear at birth or in early childhood. Vascular tumors constitute a vast number of lesions with different clinicopathological features and with varied prognoses ranging from benign, intermediate, and malignant [4].

The heterogeneous clinical morphology of the vascular malformations and tumors poses a significant problem in clinical diagnosis and necessitates the pathological examination to confirm the diagnosis. A few vascular lesions like PG, hemangioma, and solitary angiokeratoma may clinically simulate amelanotic malignant melanoma. Furthermore, atypical clinical presentation and progression similar to malignancy can pose more difficulty in the diagnosis of vascular lesions.

Dermoscopy has significantly increased the prebiopsy diagnostic accuracy of cutaneous lesions by demonstrating correlates of the lesions’ pathological features. As a supplementary diagnostic tool, it was useful in differentiating malignant melanoma from vascular tumors; the former demonstrating pigment network and melanoma-specific features while the latter shows lacunae [5, 6]. Chuh et al. found that clinical and dermatoscopic examinations diagnosed significantly more patients with vascular skin lesions than clinical examinations alone (risk ratio: 1.36; 95% confidence interval: 1.10-1.67) and the detection rate increase was significant for cherry angiomas (p < 0.001), telangiectasias (p < 0.01) and spider angiomas (p < 0.01) [7].

Most dermoscopic studies or reports on vascular malformations and tumors focused on describing the vascular features. There is sparse dermoscopic data in differentiating them from each other [1, 2]. The pathologic distinction between various vascular malformations and tumors depends upon the type of the vessels, location, and proliferation pattern. The present study evaluated the dermoscopic findings of vascular malformations and tumors based upon the dominant vascular dermoscopic features, which represent the main pathologic finding. The primary chromophores for different vascular features are hemoglobin, hemosiderin, and bilirubin. Under dermoscopy, oxygenated, deoxygenated, and oxidized hemoglobin appear red, blue, and black, respectively. The bilirubin (from extravasated erythrocytes) imparts a yellow color while hemosiderin produces a gray to blue-gray color (due to localization in the dermis).

**Figure 1:**
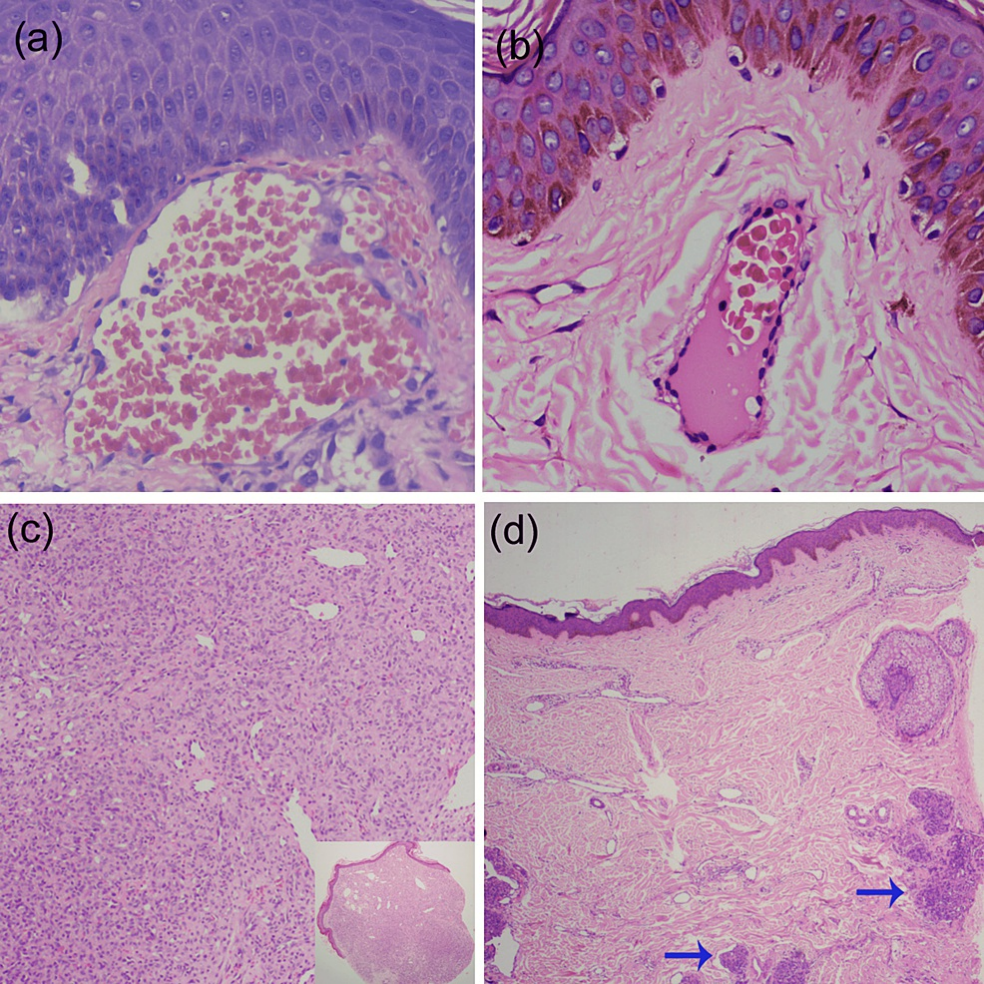
Histopathological features of vascular malformations Figure 1: (a) Solitary grossly dilated and congested ectatic vessels that represent vascular lacunae (H & E, X400). (b) Solitary dilated capillary in the papillary dermis represents a red globule (H & E, X400). (c) Diffuse endothelial proliferation with or without lumen formation represents the red structureless area (H & E, X100). (d) The deeper location of vascular tufts (arrows) in the case of a tufted angioma will not display any vascular structure (H & E, X50).

The factors that determine various dermoscopic structures in vascular tumors/malformations are the nature of the vascular lesions (proliferating versus ectatic vessels), the diameter of vessels, the arrangement of vessels, and the contents in the vessels’ lumen. We tried to demarcate between a red globule and lacunae as lacunae represent grossly dilated and congested solitary capillaries in the upper dermis and are seen in angiokeratoma. In contrast, globules represent solitary dilated blood vessels in the upper dermis (blue-gray globule and large blue-gray ovoid nest as in BCC). The red structureless area corresponds to diffuse capillary or endothelial proliferation with or without lumen formation. The location of the vascular tufts in the deep dermis will not display any vascular structures (Figure 1).

Vascular malformations

**Figure 2:**
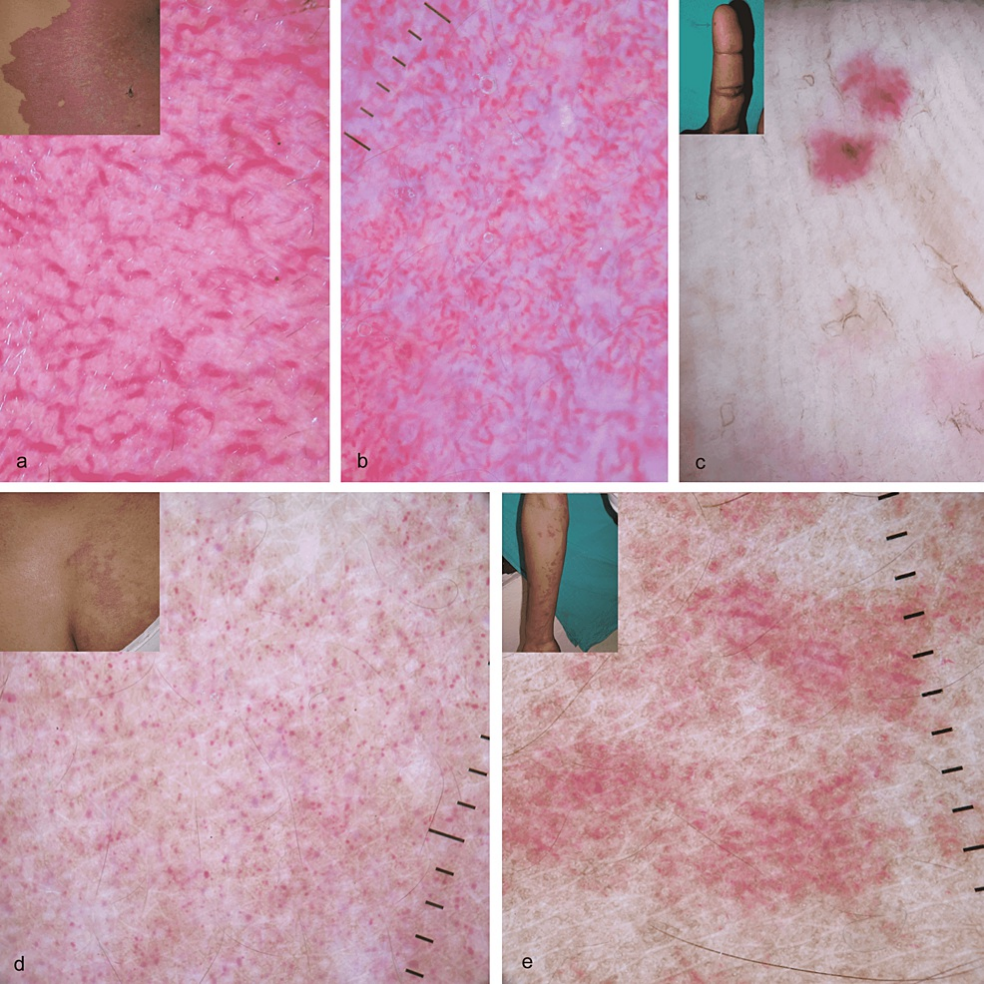
Dermoscopic features of vascular malformations (polarized mode, DermLite DL4, 10X) Figure 2: (a) Dermoscopy shows predominantly sharply-marginated linear irregular vessels in the case of port-wine stain. Note the absence of vascular dots, globules, and lacunae (inset shows port-wine stain on the trunk). (b) Dermoscopy of an advanced plaque shows linear irregular vessels separated by a white structureless area in the case of port-wine stain. (c) Dermoscopy shows ill-defined red structureless areas in the case of hereditary hemorrhagic telangiectasia (inset shows telangiectatic papule (arrow) on the finger in the case of hereditary hemorrhagic telangiectasia). (d) Dermoscopy shows predominantly red dots and globules in the case of unilateral nevoid telangiectasia. Note the absence of lacunae and structureless area (inset shows unilateral nevoid telangiectasia on the abdomen). (e) Dermoscopy shows linear irregular vessels with blurred margins in the case of angioma serpiginosum (inset shows angioma serpiginosum in a Blaschko-linear distribution on the right upper limb).

Dermoscopic features of PWS vary according to the thickness or duration of the lesions. The consistent features in our series were linear irregular vessels or linear serpentine vessels. The vessels had variable diameters and sharp margins and were arranged in parallel or haphazardly (Figure 2A). In addition, lacunae were absent, and red dots and globules were sparsely noted, mainly in the early macules. As described before, the advanced plaque showed linear irregular vessels separated by white structureless areas (Figure 2B). In the early stage, isolated dilated papillary dermal capillaries correspond to the red dots and globules, and the proliferating blood vessels parallel to the epidermis correspond to linear irregular vessels in the advanced stage. We did not observe gray-whitish veils, brown dots, or purplish vessels [8]. Dermoscopic features of HHT described are small red clods and thick serpentine and linear vessels with a somewhat radial arrangement on a pinkish background [9]. The two cases in this series showed irregular pink to red structureless areas and occasional red dots (Figure 2C). We noticed mainly red dots and globules in cases of unilateral nevoid telangiectasia (Figure 2D) in contrast to prior described red tortuous capillaries with a reticulated appearance. The dermoscopic features of AS were dominated by short linear and linear irregular vessels of variable diameter that had ill-defined margins/blurred margins (Figure 2E) and were arranged in a haphazard arrangement. Additional dermoscopic features described before are hairpin-like and comma vessels [10].

**Figure 3:**
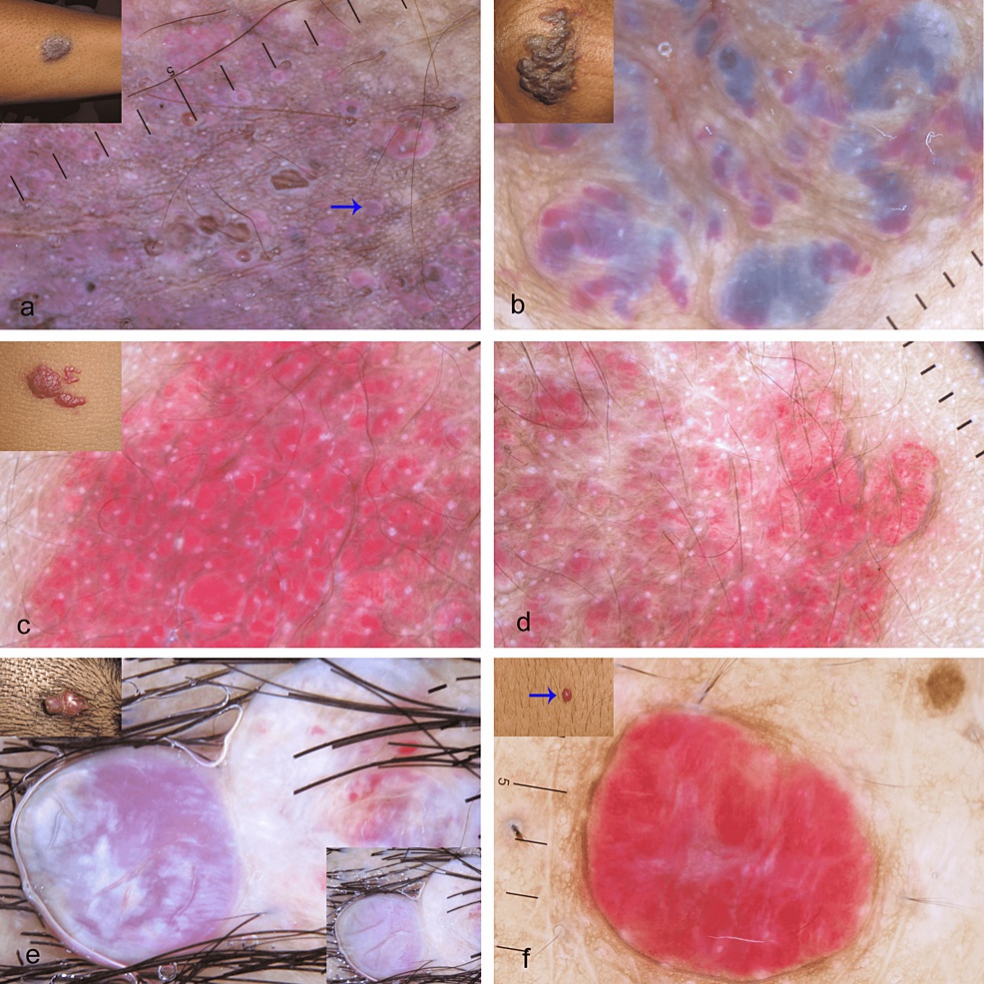
Dermoscopic features of vascular malformations and tumors (polarized mode, DermLite DL4, 10X) Figure 3: (a) Dermoscopy shows predominant red to reddish-purple lacunae (arrow) in the case of angiokeratoma. Note the absence of vascular dots and vascular structureless area, and the presence of occasional vascular globule (inset shows angiokeratoma on the forearm). (b) Dermoscopy shows multiple blue, red, and bluish-red lacunae and structureless areas in the case of venous malformation (Inset shows venous malformation of the scrotum). (c) and (d) Dermoscopy shows a mixed vascular pattern: red dots, globules, lacunae, and structureless area in the case of infantile hemangioma (inset shows infantile hemangioma on the trunk). (e) Dermoscopy shows a red structureless area and shiny white structures under polarized mode in the case of pyogenic granuloma (Inset shows pyogenic granuloma on the scalp). The prominence of white color under nonpolarized mode (inset, right lower side). Note the absence of red dots, globules, and lacunae. (f) Dermoscopy shows a red structureless area and white lines in the case of cherry angioma. Note the absence of red dots, globules, and lacunae (inset shows cherry angioma (arrow) on the trunk).

In line with the prior report, in all subtypes of AK, the prominent dermoscopic features were red, reddish-purple to brown-black lacunae (Figure 3A) [11, 12]. The red to reddish-purple lacunae represent dilated and congested capillaries and the brown-black lacunae represent thrombosed capillaries. A solitary case showed a white network with the network hole occupied by red lacunae. A single case of venous malformation in the index study showed red, blue, reddish-blue, and reddish-purple lacunae and structureless areas (Figure 3B). Dermoscopic features described for glomuvenous malformation include red-blue lacunae, blue-white veil, whitish areas of fibrosis, and a form of arborizing vessels. In our series, a plaque-type glomuvenous malformation showed ill-defined bluish-pink structureless areas, occasional red dots and globules, and linear vessels, while the papular type revealed a red homogenous area [13]. Dermoscopy is quite useful for the diagnosis of venous lakes, especially to rule out melanoma. Asian and Western studies report reddish-blue lacunae, globules/clods, and homogenous blue areas as dermoscopic features [14]. Blue to bluish-purple structureless areas were the dermoscopic findings in our case without any lacunae.

Vascular tumors

In the present study, a red to reddish-white structureless area was the common dermoscopic feature in all cases of superficial IH. In addition, red dots, globules, and lacunae were also noted. We termed the combination of these vascular structures a mixed pattern (Figures 3C-3D). In contrast to prior reports, we did not observe comma-like, wavy, and red linear vessels [15]. In this series, a solitary deep IH had linear irregular vessels and white to bluish-white structureless areas. The features described prior are polymorphous vascular structures with red linear and red dilated vessels, polymorphous vascular patterns; red globular vessels, red circulated vessels, red wavy vessels, red linear vessels, and red dilated vessels [2]. The constant dermoscopic feature in PG was a red to reddish-white structureless area (Figure 3E), representing proliferating endothelial cells with or without lumen formation. A white line and shiny white structures were observed in a few cases of PG. A single case showed a multicolored pattern under polarized mode. A similar dermoscopic pattern was observed in the cases of cherry angioma; red to reddish-white structureless areas were the constant feature (Figure 3F), and less commonly observed features were red globules, lacunae, linear vessels, and white lines. However, cherry angiomas did not show blood spots and crusting in contrast to PG.

**Figure 4:**
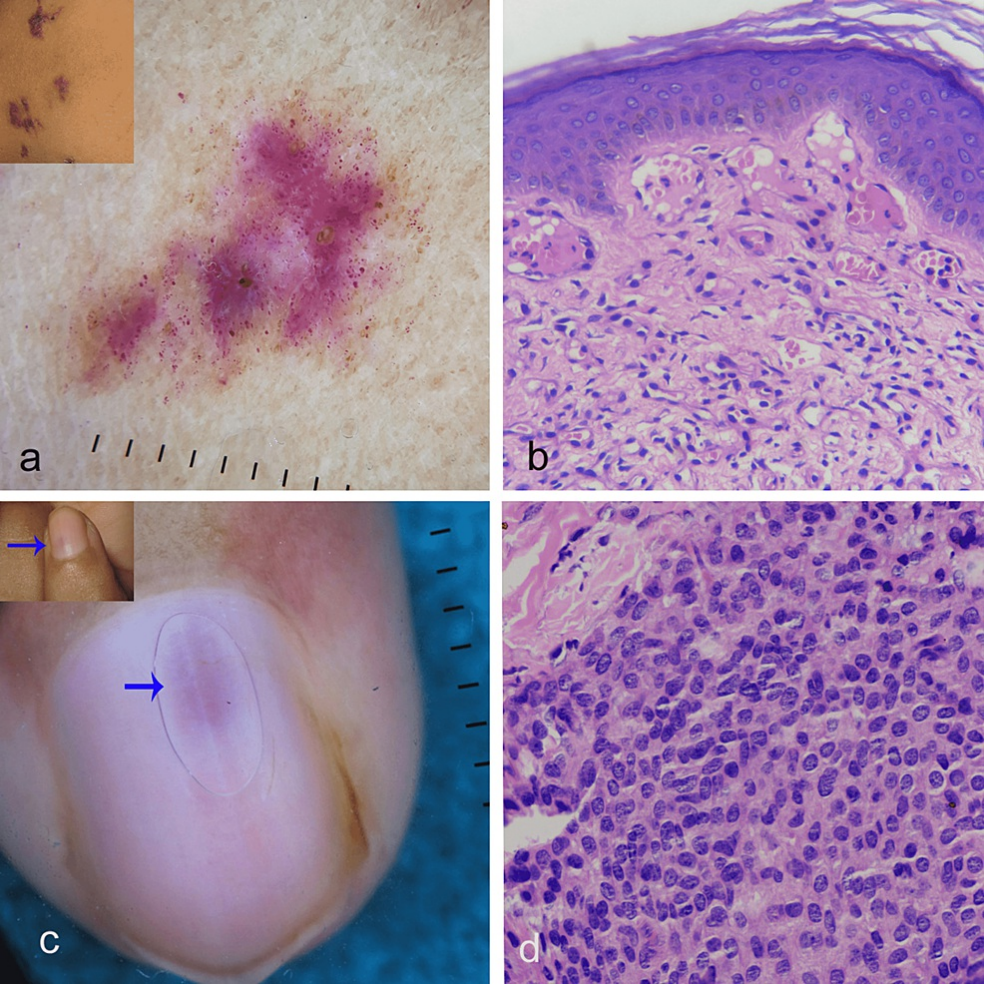
Dermoscopic and histopathological features of vascular tumors Figure 4: (a) Dermosopy (Polarized mode, DermLite DL4, 10X) shows prominent red dots, globules, and structureless areas. Note the presence of keratotic plug and the absence of lacunae (Inset shows verrucous hemangioma on the thigh). (b) Diffuse capillary proliferation in the upper dermis in the case of verrucous hemangioma. Note the absence of grossly dilated and congested vessels as seen in angiokeratoma (H & E, X400). (c) Dermoscopy shows (Polarized mode, DermLite DL4, 10X) ill-defined pink structureless area in the case of subungual glomus tumor (Inset shows subungual (arrow) glomus tumor). (d) The proliferation of cuboid glomus cells (H & E, X400)

The dermoscopic features of VH can vary depending upon the stage of the disease and the resulting hyperkeratosis [16]. Two early VH in this series had predominantly red to reddish-purple dots, globules, and structureless areas (Figure 4A). In addition, comedo-like openings with keratotic plugging were the constant nonvascular feature. Advanced lesions may show prominent hyperkeratosis and, in addition, hinder the visibility of vascular structures. One of the cases in this study showed prominent shiny white structures (SWSs) comprising rosette, shiny white lines, clod, and structureless areas. The SWSs correspond to the compact hyperkeratosis. In contrast to AK, we did not observe red lacunae in VH cases, as the vessels in the upper dermis (Figure 4B) were not grossly dilated and ectactic as in AK. However, a biopsy is needed to demonstrate the proliferating vessels in the lower dermis and subcutis, the hallmark feature of diagnosis VH.

Dermoscopy of tufted angioma reveals a typical pigment network overlying very slight pinkish telangiectasias, numerous tiny red lacunae separated by thin, whitish linear septa. Our single case had a cerebriform pattern and a faint skin-colored to the pinkish-white structureless area. This is due to the deeper location of vascular tufts in our case and the overlying epithelial hyperplasia [17].

A consistent feature of dermoscopy of extra digital glomus tumor includes structureless, homogeneous, patternless lesions, which we also observed. Other features like a central purple area with a peripheral whitish homogeneous area; yellow-to-white background; and multiple telangiectasias have been described [18, 19]. The consistent dermoscopic feature in our three subungual glomus tumors was an ill-defined pink structureless area (Figures 4C-4D]. In an appropriate clinical setting, this feature may be helpful in the diagnosis of subungual glomus tumors [20]. 

The color of vascular structures appeared reddish-white under the nonpolarized mode, compared to red color under polarized mode. Under nonpolarized mode, the margins of various vascular structures became more sharply defined than in polarized mode.

**Figure 5:**
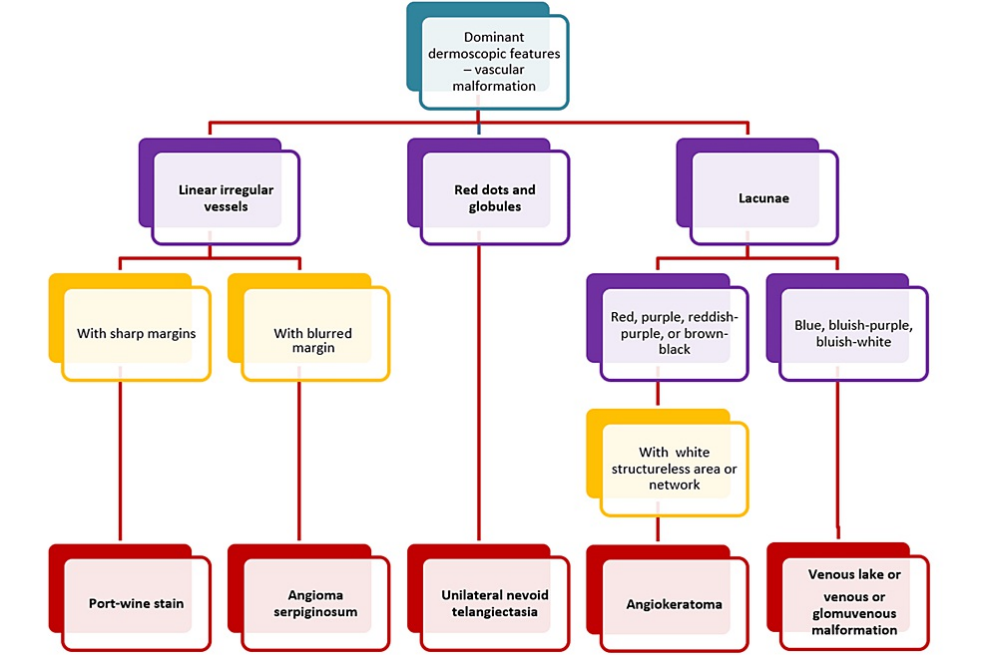
A proposed diagnostic algorithm for vascular malformations. A proposed diagnostic algorithm for vascular malformations.

**Figure 6:**
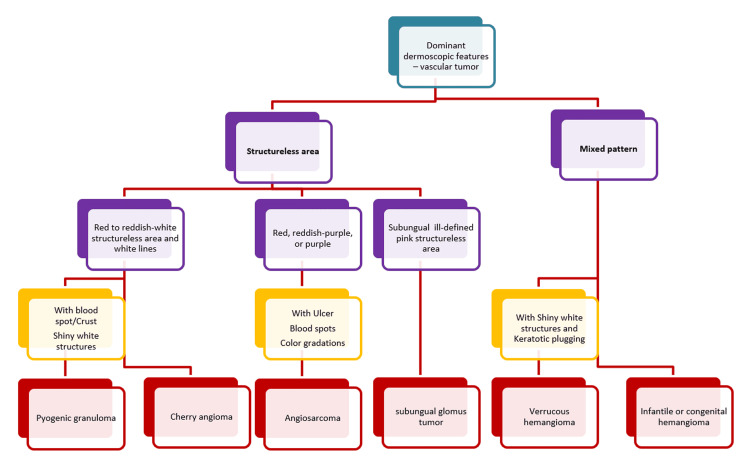
A proposed diagnostic algorithm for vascular tumors A proposed diagnostic algorithm for vascular tumors

This study classified and described the vascular dermoscopic features based on the nature and pattern of cutaneous vascular structures. In addition, we propose a diagnostic algorithm based upon the dominant dermoscopic vascular feature and its color (Figures 5-6), which needs to be validated in the future owing to the small number of cases in each group in our study. 

The various limitations of this study were the small sample size, data from a single center, and the absence of a comparison group.

## Conclusions

In conclusion, we describe the dermoscopic features of vascular malformations and tumors based on dominant vascular features. The dominant vascular dermoscopic features are distinct in different vascular lesions with few exceptions. These include linear irregular vessels, red dots and globules, lacunae and structureless areas of various colors, and a mixed pattern. In addition, the color of the vessels and the altered blood inside the vessels’ lumen provide additional clues to the diagnosis. However, our study’s interpretation was limited by the small sample size. A multicentric study with a large sample size will help verify our findings.
